# Characterization of a highly conserved MUC5B-degrading protease, MdpL, from *Limosilactobacillus fermentum*

**DOI:** 10.3389/fmicb.2023.1127466

**Published:** 2023-02-28

**Authors:** Fredrik Leo, Gunnel Svensäter, Rolf Lood, Claes Wickström

**Affiliations:** ^1^Department of Oral Biology and Pathology, Faculty of Odontology, Malmö University, Malmö, Sweden; ^2^Genovis AB, Lund, Sweden; ^3^Department of Clinical Sciences Lund, Division of Infection Medicine, Faculty of Medicine, Lund University, Lund, Sweden

**Keywords:** MUC5B, *Limosilactobacillus fermentum*, O-glycan, oral microbiota, mucin degradation, protease

## Abstract

MUC5B is the predominant glycoprotein in saliva and is instrumental in the establishment and maintenance of multi-species eubiotic biofilms in the oral cavity. Investigations of the aciduric *Lactobacillaceae* family, and its role in biofilms emphasizes the diversity across different genera of the proteolytic systems involved in the nutritional utilization of mucins. We have characterized a protease from *Limosilactobacillus fermentum*, MdpL (Mucin degrading protease from *Limosilactobacillus*) with a high protein backbone similarity with commensals that exploit mucins for attachment and nutrition. MdpL was shown to be associated with the bacterial cell surface, in close proximity to MUC5B, which was sequentially degraded into low molecular weight fragments. Mapping the substrate preference revealed multiple hydrolytic sites of proteins with a high O-glycan occurrence, although hydrolysis was not dependent on the presence of O-glycans. However, since proteolysis of immunoglobulins was absent, and general protease activity was low, a preference for glycoproteins similar to MUC5B in terms of glycosylation and structure is suggested. MdpL preferentially hydrolyzed C-terminally located hydrophobic residues in peptides larger than 20 amino acids, which hinted at a limited sequence preference. To secure proper enzyme folding and optimal conditions for activity, *L. fermentum* incorporates a complex system that establishes a reducing environment. The importance of overall reducing conditions was confirmed by the activity boosting effect of the added reducing agents L-cysteine and DTT. High activity was retained in low to neutral pH 5.5–7.0, but the enzyme was completely inhibited in the presence of Zn^2+^. Here we have characterized a highly conserved mucin degrading protease from *L. fermentum*. MdpL, that together with the recently discovered O-glycanase and O-glycoprotease enzyme groups, increases our understanding of mucin degradation and complex biofilm dynamics.

## Introduction

By formation of covalently bonded polymeric structures, gel-forming mucins constitute the framework of the protective matrix covering all epithelial surfaces throughout the body (Bell et al., 1985; Sellers et al., 1988). These 2–40 MDa oligomeric glycoproteins which contain O-glycans that contribute to as much as 80% of the total mass, have many functions including: lubrication of the epithelial surface, filtration of nutrients, protection from pathogens, and also mitigation of mechanical and chemical stresses (Wickström and Svensäter, 2008; Zaretsky and Wreschner, 2013b). Different oligomeric mucins are found at different sites, e.g., MUC2 is the major gel-forming mucin in the small intestine and colon whereas MUC5B is the predominant mucin of the oral cavity, the respiratory tract, and the endocervix (Wickström et al., 1998; Thomsson et al., 2002; Zaretsky and Wreschner, 2013b). Being a key constituent of saliva, MUC5B promotes the initial adhesion of early microbial colonizers and also comprises the main nutrient source for oral bacteria (Kolenbrander, 2011; Jakubovics, 2015). MUC5B functionality is significantly regulated by glycosylation, and O-glycans play important roles regarding viscosity and adhesivity variations, with the 292 O-linked chains (40% sialylated) found on average per 2–2.5 MDa mucin monomer (Thomsson et al., 2002; Zaretsky and Wreschner, 2013a). An average neutral O-glycan chain consists of 13 monosaccharides, whereas the sialylated and the sulfated chains have 17 and 41, respectively (Thomsson et al., 2002). MUC5B is differently glycosylated even within the same gland, making it highly diverse in terms of density, sulfation, and sialylation, thus justifying in part the biofilm variation at different oral sites (Shori et al., 2001).

*Limosilactobacillus fermentum* is commonly isolated from the gastrointestinal tract where it is suggested to have a probiotic function with immunomodulatory, anti-inflammatory, and anti-oxidative effects (Song et al., 2000; Tulumoglu et al., 2013; Archer and Halami, 2015; Park et al., 2015; Garcia-Castillo et al., 2019). *Limosilactobacillus fermentum* together with other *Lactobacillaceae* (historically known as the *Lactobacillus* genera) species is also part of the complex oral microbiome that colonize teeth as multi-species biofilms (Kilian et al., 2016). Lactobacilli comprise less than 1% of the total cultivable microbiota in the healthy mouth but its probiotic role is not clear. The proportion and prevalence of lactobacilli increase in severe caries lesions, and infected dentine has a high proportion of lactobacilli and other acid-tolerating and proteolytic microorganisms. Contrary to the aetiology of most infections, no single bacterial species is uniquely associated with the development of dental caries (Marsh, 1994). Instead, it is believed that the disease is a consequence of a microbially driven sharp pH drop of the dental biofilms, resulting in a dysbiotic shift of the resident dental microbiota toward acid-tolerating microorganisms, including the lactobacilli. The role of *L. fermentum* in the mouth is debated, and studies suggest that the bacterium have both positive (Terai et al., 2015; Mann et al., 2021) and negative health effects (Marchant et al., 2001; Lapirattanakul et al., 2020). Salivary MUC5B serves as the most important nutrient supply for the oral microbiome, satisfying the microbial community demand for carbon and nitrogen (Wickström et al., 2009a). Owing to the structural complexity of MUC5B, oral streptococci can only partially degrade glycans (de Jong and van Der Hoeven, 1987; van Der Hoeven et al., 1990). Hence, exhaustive degradation can be accomplished only by the cooperation of multi-species biofilm consortia (Wickström et al., 2009b). The combination of bacteria in the consortia cannot be random since their enzymatic degradation pathways need to be complementary. Therefore, it is more likely than not that the proteolytic activity of lactobacilli is essential in the degradation of MUC5B.

The enzymatic mucin degradation hypothesis maintains that degradation is dependent on glycosidases, O-glycanases, proteases, and O-glycoproteases acting in concert (Shon et al., 2021). The O-glycan-rich domains are thought to be resistant to proteolysis, e.g., to trypsin and elastase, implying that the aforementioned glycosidases and O-glycoproteases finalize the hydrolysis (Shon et al., 2021). The glycosidases habitually expressed by early colonizers, i.e., neuraminidase, α-fucosidase, β-N-Acetylglucosaminidase, α-galactosidase, β-galactosidase, and β-N-Acetylgalactosaminidase, hydrolyze monosaccharide moieties usually located on MUC5B O-glycans (van Der Hoeven et al., 1990). O-glycanases are typically the initiators of mucin degradation, by the endo-acting hydrolysis of polyLacNAc (Galβ1-4GlcNAc)_n_ structures in oligosaccharide side chains (Crouch et al., 2020). The newly discovered O-glycoprotease enzyme group, including the enzymes OgpA, CpaA, ImpA, StcE, ZmpB, and BT4244, are characterized by their ability to hydrolyze the protein backbone, dependent on the particular proximal O-glycan (Shon et al., 2021). The action of these enzymes constitutes the only mucin degrading pathways known as of today.

A study investigating the proteolytic activity of a clinical *L. fermentum* strain isolated from dental plaque, uncovered increased expression of several chaperones and a putative O-sialoglycoprotein endopeptidase (OSGP), thus accounting for the protein content of surface-associated biofilms generated by growth in a MUC5B environment (Wickström et al., 2013). A characterization of an OSGP enzyme from *Pasteurella haemolytica* indicated activity toward substrates decorated with sialylated O-glycans (Mellors and Lo, 1995), strongly suggesting that the enzyme has the potential of hydrolyzing mucin. However, the natural habitat of the two bacteria differs, as does the enzyme primary structure, indicating that the *L. fermentum* and *P. haemolytica* enzymes have dissimilar features. In this work, we have investigated OSGP from *L. fermentum* and characterized its physicochemical optima, glycan dependency, substrate specificity, cellular localization, and interactions with MUC5B. Due to its ability to act on mucin, the protein will be denoted as MdpL (Mucin Degrading Protease L) throughout the article.

## Materials and methods

### Sequence homology of bacterial enzymes

Sequence homologies of MdpL (GenBank accession number: WP_057727250.1) from different *L. fermentum* strains as well as related species were calculated using ClustalOmega. A table with all included bacterial species and their respective GenBank accession number can be found in Supplementary Table 1. A phylogenetic tree was constructed using MacVector (MacVector Inc., United States) based on neighbor-joining and bootstrap analysis. The values on each branch represent the estimated confidence limit (%) for the position of the branch.

### Recombinant expression and production of MdpL inclusion bodies

Codon optimized gene constructs representing the whole MdpL protein (GenBank accession number KRN17993.1) containing no predicted signal peptide were inserted into a pET21(a) plasmid (Genscript, United States). KRN17993.1 was selected due to the absence of the redundant seven amino acid N-terminal sequence, but with a high identity to WP_057727250.1 (99.7%). It was recombinantly expressed in *Escherichia coli* BL21(DE3) STAR cells as a fusion protein with a C-terminal Gly-Ser-Gly (GSG) linker and 6 × His-tag. The FASTA sequence of MdpL can be found in Supplementary Figure 1. Bacteria were cultured (37°C, 220 rpm) in Luria Broth until OD_600_ reached 0.6 and the cells were induced with 1 mM IPTG. Protein expression was continued for 5 h (37°C, 220 rpm), before collecting and freezing cells (−20°C).

### Purification of MdpL from inclusion bodies

Frozen cells with MdpL inclusion bodies were dissolved in BugBuster (Merck Millipore, United States) with end-over-end mixing (15 min, RT) after which the cellular material as well as the inclusion bodies were collected. This step was repeated with vortexing before adding lysozyme to the cell mixture (5 min, RT) with another round of vortexing. Diluted BugBuster (1:10) was added, gently vortexed (1 min, RT) and cells were collected, and the procedure was repeated once prior to storage (−20°C). The thawed pellets were dissolved in a denaturing buffer (TBS pH 7.6, 8 M Urea, 40 mM DTT), mixed (2 h, RT) and then pH-adjusted to pH 4.0 using 1 M hydrochloric acid. Samples were applied to a PD10 column (Cytiva Lifesciences, Sweden) equilibrated with a 10 mM HCl solution. The protein concentration was measured using Nanodrop (Spectrophotometer-ND1000, Thermo Scientific, United States) before refolding the enzyme according to the method described by Watt et al. (1997). Briefly, denatured MdpL was incubated with protein disulfide isomerase (Merck Millipore) and reduced/oxidized glutathione (Merck Millipore) in 50 mM sodium phosphate, pH 7.5 (18 h, 30°C). A schematic overview including a brief explanation of all steps can be found in Supplementary Figure 2.

### Physicochemical characterization of MdpL

The conditions allowing optimal MdpL activity was assessed. The effect of temperature (29–45°C), cations/EDTA (2 mM CaCl_2_, MgCl_2_, ZnCl_2_, EDTA), NaCl (0–500 mM), and reducing agents (L-cys (10–100 mM), DTT (20–100 mM)) on MdpL activity toward Etanercept (Pfizer Inc., United States) was determined using SDS-PAGE with densitometric quantification using Image Lab software (Version 6.0, Bio-Rad laboratories, United States). The pH-optimum was determined using 100 mM sodium acetate buffer, pH 4.5–5.5, and 100 mM sodium phosphate buffer, pH 6.0–8.0, followed by densitometric quantification. The relative activity was calculated by dividing the separate measured activity values with the maximum activity, whereas the activity after addition of L-cys or DTT was concluded by calculating the percentage of hydrolyzed substrate. In all assays, MdpL was incubated with Etanercept (4 h, 37°C) in technical triplicates.

### Protease inhibition test

Potential inhibition of the proteolytic activity of MdpL was studied with a panel of protease inhibitors: AEBSF, ALLN, antipain, bestatin, chymostatin, E64, EDTA-Na_2_, leupeptin, pepstatin, phosphoramidon, and PMSF (G-Biosciences, Geno Technology Inc., United States) following the manufacturer’s instruction. MdpL inhibition was analyzed by gel electrophoresis using Etanercept as the substrate.

### General proteolytic activity

The general proteolytic activity of MdpL was determined by incubation of a substrate derived from casein (EnzChek Protease Assay Kit, Thermo Fisher Scientific) in 0.1 M Tris, pH 7.0, which was analyzed using a fluorescence-based assay, where RgpB (GingisREX®, Genovis AB, Sweden), and IdeS (FabRICATOR®, Genovis AB) were used as positive and negative controls, respectively. 20 mM L-cys was included in the reaction for MdpL and RgpB.

### Immunoglobulin specificity and O-glycan dependency test

The immunoglobulin specific activity was measured with densitometric quantification from reduced SDS-PAGE, after MdpL incubation with IgG (1,2,4), pIgA, CTLA-4-IgG1 (Abatacept), TNFR-IgG1 (Etanercept), and Fetuin (18 h, 37°C) in 0.1 M sodium phosphate, pH 6.5, with 20 mM L-cys. Investigating the O-glycan dependence/independence of MdpL, the latter four substrates were also deglycosylated using α2-3,6,8 neuraminidase (SialEXO®, Genovis AB), endo-α-N-acetylgalactosaminidase (OglyZOR®, Genovis AB), and α-N-acetylgalactosaminidase (GalNAcEXO™, Genovis AB) prior to MdpL digestion (18 h, 37°C). Humanized IgG1 Trastuzumab (Roche, Switzerland), human IgG2 Panitumumab (Amgen, United States), human IgG4 Nivolumab (Bristol-Myers Squibb, United States), native pIgA purified from plasma (Calbiochem, United States), fusion protein Abatacept (Bristol-Myers Squibb), fusion protein Etanercept (Pfizer Inc.), and fetal calf serum Fetuin (NEB, United States) were used.

### LC–MS/MS analysis of peptide hydrolysis

The peptides Drosocin (Anaspec, United States), H2686, H8390, Amyloid β (10–20), Neuromedin U-25, MOG (Bachem, Switzerland), and Insulin Chain B Oxidized (IOB; Sigma, United States) were incubated with MdpL (18 h, 37°C) in 0.1 M sodium phosphate, pH 6.5, with 20 mM L-cys to detect any preferential sites permitting MdpL hydrolysis. Fragments were separated using LC (Agilent Technologies 1,290 Infinity, C18) and analyzed by MS/MS (Bruker Impact II). MdpL hydrolysis sites were mapped using the Bruker Compass Data Analysis 5.2 and BioPharma Compass 4.0 software. Since the generated peptide ions have different flight characteristics during an ESI-TOF-MS run, the prevalence of amino acids was not based on area under the curve calculations. A sequence logo (WebLogo 3, University of Berkeley) illustrating the most occurring amino acid +/− three positions from the MdpL hydrolytic site was created by entering the 89 detected high score (>30) peptides generated from the hydrolysis of Neuromedin U-25, MOG, and Insulin Chain B Oxidized. Drosocin, H2686, H8390, and Amyloid β were not hydrolyzed by MdpL and thus were not included in the data acquisition. The mass spectrometry proteomics data have been deposited to the ProteomeXchange Consortium *via* the PRIDE (Perez-Riverol et al., 2021) partner repository with the dataset identifier PXD039681.

### LC–MS/MS analysis of MUC5B degradation

Human MUC5B purified from whole saliva as previously described (Wickström and Svensäter, 2008) was incubated with MdpL (20 h, 37°C) and 20 mM L-cys in 50 mM sodium phosphate, pH 7.5. The enzyme was inhibited using 2 mM Zn^2+^, before running the sample over an PNGase F Immobilized column (Genovis AB). The sample was incubated with Lys-C (Thermo Scientific) in 0.1 M Tris pH 7.6 (5% SDS; 18 h, 37°C) and the fragments were separated using LC (Evosep ONE, nano-UHPLC, C18), and analyzed by MS/MS (Thermo Scientific Q Exactive HF-X Hybrid Quadropole-Orbitrap). The MdpL digestion sites were mapped using Xcalibur 4.1 (Thermo Scientific) for data acquisition and MaxQuant 1.6 (Max Planck Institute of Biochemistry, Germany) for data analysis. The mass spectrometry proteomics data have been deposited to the ProteomeXchange Consortium *via* the PRIDE (Perez-Riverol et al., 2021) partner repository with the dataset identifier PXD039681.

### Degradation of MUC5B analyzed by SDS-PAGE

To further confirm the activity of MdpL toward MUC5B, the enzyme was employed in reactions with reduced human saliva MUC5B in 0.1 M sodium phosphate, pH 6.5, with 20 mM L-cys using different reaction times, *viz.* 1, 2, 24 h, at 37°C. All reactions were quenched at one selected timepoint, at which they were heated (10 min, 70°C), mixed with LDS and then loaded onto a 4–12% Bis-Tris gel (Thermo Scientific) in an extended run (1 h, 200 V constant). Double staining with Coomassie and Silver (Sigma), was followed by image analysis using ImageLab (Bio-Rad laboratories) immediately after band development.

### MUC5B degradation analyzed by Western Blot

To confirm the MdpL-degradation of intact or fragmented MUC5B, the reaction described above for the SDS-PAGE analysis was employed, using a 24 h reaction time. Immediately after the electrophoresis, the gel was transferred to a PVDF membrane using a Trans-Blot turbo pack (Bio-Rad) and then blocked with 5% (w/v) skim milk (Oxoid, United States) in TBST (20 mM Tris, 137 mM NaCl, 0.05% Tween-20) for 1 h (RT). MUC5B was detected (1 h, RT) using an anti-serum, LUM5B-14 (diluted 1:500 in skim milk/TBST), raised against a synthetic peptide with the sequence CRAENYPEVSIDQVGQVL present in the third Cys domain of MUC5B. The membrane was then incubated (1 h, RT) with a horseradish peroxidase-conjugated polyclonal goat anti-rabbit antibody (Dako, Denmark) diluted 1:2500 in skim milk/TBST before visualizing bands using the Pierce ECP Western Blot substrate (Thermo Fisher Scientific) and imaging in an iBright Imaging System (Thermo Fisher Scientific).

### Determining the cellular localization of MdpL

The cell localization of MdpL was evaluated by analyzing the content in three different samples: papain-digested cells (surface associated: cell wall-attached), sonicated cells (lysate: intracellular and cell wall-attached), and the bacterial supernatant (extracellular). A clinical strain of *L. fermentum* was cultivated in de Man, Rogosa, and Sharpe (MRS) broth (17 h, 37°C) after which the cells and supernatant were separated and stored in the freezer (−20°C). One cell fraction was dissolved in 0.1 M Tris, pH 7.6, (5% SDS) before being sonicated for release of intracellular proteins (6 × 5 min with an equally long pause between each step, 70% efficiency, kept on ice) after which the supernatant was collected. A second cell fraction was incubated with 5 μg papain (Sigma) and L-cys (1 h, 37°C), prior to inhibiting further hydrolysis with leupeptin (Sigma) and collecting the supernatant. The *L. fermentum* supernatant was concentrated and re-buffered in PBS before being analyzed. The three samples were incubated with trypsin (Promega, United States) and recognized MdpL peptide sequences were identified and quantified from a database search with MaxQuant 1.6 against a *L. fermentum* genome.

## Results

### High MdpL conservation within the *Limosilactobacillus fermentum* species and high homology with early biofilm colonizers

**Figure 1:**
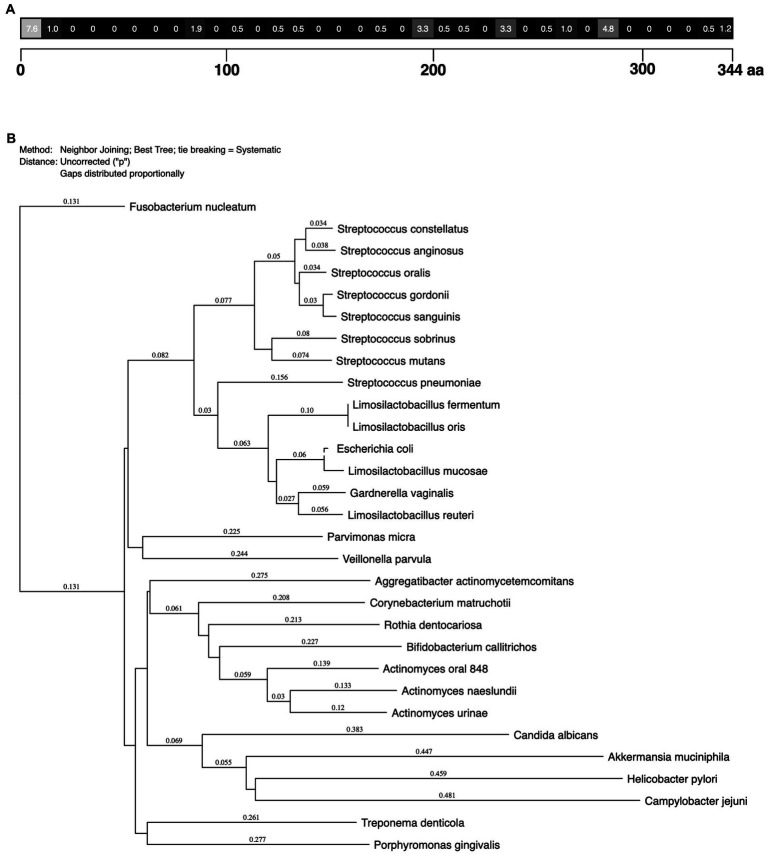
*Limosilactobacillus fermentum* MdpL alignment and the degree of protein sequence conservation. **(A)** Numbers display the percentage (%) of changed amino acids in a block of 10 amino acids, compared to MdpL from one selected *L. fermentum* strain (GenBank: WP_057727250.1). **(B)** Phylogenetic tree of MdpL and related proteins from other species. The number above the branch points denote the confidence level of the relationship of the paired sequences determined by boot strap statistical analysis. The tree is not drawn to scale.

MdpL conservation across various bacteria was investigated using different bioinformatics methods (Figure 1). A low incidence of changed amino acids in the MdpL sequence indicated that the MdpL primary structure was highly conserved in the *L. fermentum* species (Figure 1A). The main difference was seen in the N-terminal part (aa 1–10), given that eight initial amino acids were missing in two strains. A majority of the other variances were single amino acid mutations, likely not affecting biological properties.

Besides the *L. fermentum* strains, MdpL was highly conserved in the *Limosilactobacillus* genus, in *Gardnerella vaginalis*, and in one strain of *E. coli*, and incidentally, the first two regularly inhabit MUC5B-rich locations (Figure 1B). Other high MdpL correspondence species were the supragingival early biofilm colonizers of the *Streptococcus* genus, including the caries-associated *Streptococcus mutans*. The subgingival late biofilm colonizers *Parvimonas micra* and *Rothia dentocariosa*, but also some supragingival *Actinomyces* species were more distantly related. The essentially unrelated *Helicobacter pylori* and *Akkermansia muciniphila*, which are found in the gut, and the periodontitis-associated *Porphyromonas gingivalis*, all lacked an MdpL-like enzyme.

### MdpL is a protease with properties apt for an oral biofilm environment

**Figure 2:**
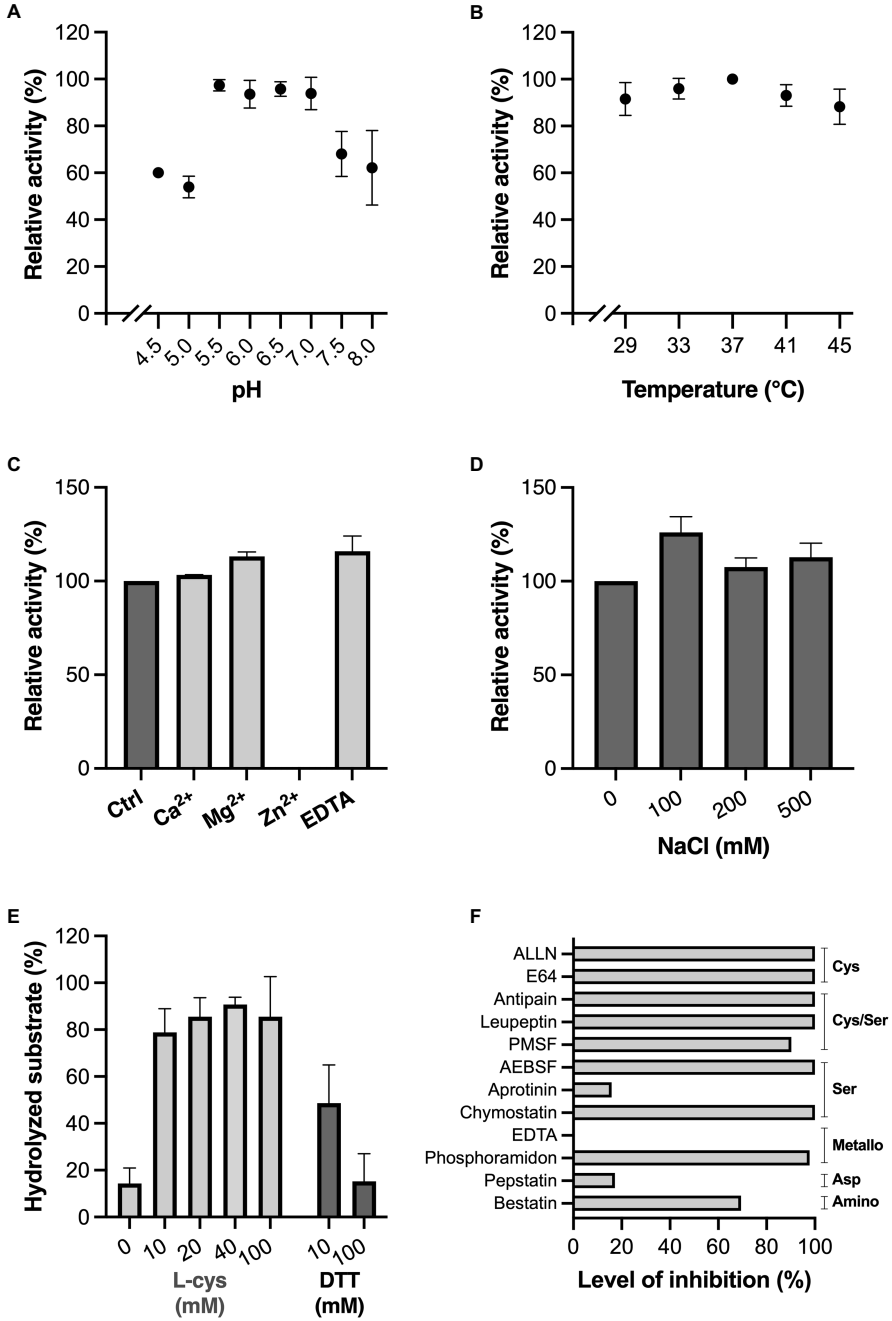
Influence of physicochemical conditions and inhibitors on MdpL activity. Effect of **(A)** pH, in the interval 4.5–8.0, **(B)** biologically relevant temperatures (29–45°C), **(C)** 2 mM Ca^2+^, Mg^2+^, Zn^2+^, and EDTA, **(D)** increasing NaCl concentrations (0–500 mM), **(E)** the reducing agents L-cys and DTT at increasing concentrations (0, 10, 20, 40, 100 mM, and 10, 100 mM, respectively), **(F)** common protease inhibitors, where the inhibition level is displayed as percentage in relation to baseline MdpL activity. Common protease families inhibited by the protease inhibitors are noted to the right of the graph. All assays were conducted as technical triplicates and relied on SDS-PAGE and densitometric quantification. Relative fluorescence values recorded from the samples were used to calculate the relative activity (%). The level of hydrolyzed substrate (%) in E is the calculated percentage of digested substrate. Data in **A–E** is plotted as mean values ± SD.

The characteristics of MdpL were determined using electrophoresis with densitometric quantification (Figure 2). Etanercept, with a mucin-like glycosylation pattern with up to 13 O-glycans and three N-glycans in each monomer, was used for the characterization tests owing to its highly homogenous nature and high resemblance to MUC5B. Since enzyme kinetics are substrate dependent, using Etanercept instead of standard soluble substrates generated more biologically relevant physicochemical data. The activity of MdpL peaked in the low to neutral pH range, i.e., 5.5–7.0, but retained 60% of the relative activity at lower (4.5) and higher pH (8.0; Figure 2A). The temperatures tested (29–45°C) did only marginally affect the activity, but an activity peak was observed at 37°C (Figure 2B). Divalent cation and EDTA effects on MdpL activity were studied by including CaCl_2_, MgCl_2_, ZnCl_2_, and EDTA in the reaction buffer. Addition of CaCl_2_, MgCl_2_, and EDTA slightly increased the Etanercept hydrolysis, while ZnCl_2_ completely inhibited the enzyme (Figure 2C). MdpL showed retained activity in all tested sodium chloride concentrations, indicating that the enzyme is not particularly sensitive to drastic changes in ionic strength (Figure 2D). Addition of the reducing agents L-cys or DTT resulted in an activity increase, already at low molar levels (10 mM), but remained low at high DTT levels (100 mM; Figure 2E). Protease inhibitors were added to the reaction buffer to investigate the protease family classification of MdpL. AEBSF, ALLN, Antipain, Chymostatin, E64, Leupeptin, and Phosphoramidon completely inhibited the MdpL activity, whereas Bestatin, and PMSF had a moderate inhibitory effect. Aprotinin, EDTA, and Pepstatin did not affect the activity (Figure 2F). Representative gels for each physicochemical test can be found in Supplementary Figures 3–7.

### O-glycan independent digestion of substrates with mucin-like properties

**Figure 3:**
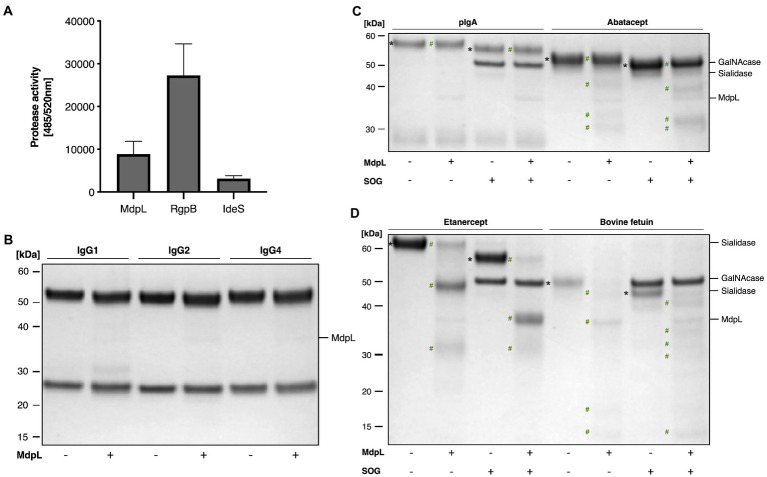
Proteolytic specificity and absence of O-glycan dependence of MdpL. MdpL activity toward **(A)** fluorescently labeled casein; RgpB (Arg-specific) and IdeS (IgG-specific) were included as controls, **(B)** human IgG subclasses, (**C,D**) O-glycoproteins with or without Core 1 O-glycans. SOG depicts the glycan hydrolyzing enzymes Sialidase, O-glycosidase, and α-GalNAcase. Assay **(A)** was conducted using the fluorescence based EnzChek Protease Assay. Protein substrates in Assays **(B–D)**, including IgG1, IgG2, IgG4, pIgA, Abatacept, Etanercept, and bovine fetuin were incubated with MdpL (37°C, 18 h) and analyzed using SDS-PAGE under reducing conditions. Intact and deglycosylated substrates (*), and MdpL-generated fragments (#). MdpL and SOG reference bands can be found in Supplementary Figure 8.

The features of MdpL were studied further using a diverse range of enzyme substrates (Figure 3). The proteolytic preference of MdpL was evaluated using a fluorometric quantification of casein hydrolysis. Wide ranging substrate hydrolysis leads to an increase in fluorescence and gets higher when a general Lys/Arg-specific enzyme such as RgpB is included in the reaction. An IgG-specific enzyme such as IdeS does not hydrolyze casein and is used as a negative control. MdpL has an intermediate activity between the controls, i.e., about 30% of that of RgpB (Figure 3A).

In order to elucidate MdpL’s potential immunomodulatory effect, the enzyme was incubated with different Immunoglobulins. Neither IgG, nor IgA (intact or deglycosylated) were significantly affected by MdpL (Figures 3B,D). In the case of two therapeutic fusion proteins relying on the IgG backbone (i.e., Abatacept and Etanercept), hydrolysis could be detected, both in the presence and absence of O-glycans (Figure 3C). The highest activity was however detected with bovine fetuin, again independent on glycosylation level (Figure 3D).

### MUC5B is proteolytically degraded by MdpL

**Figure 4:**
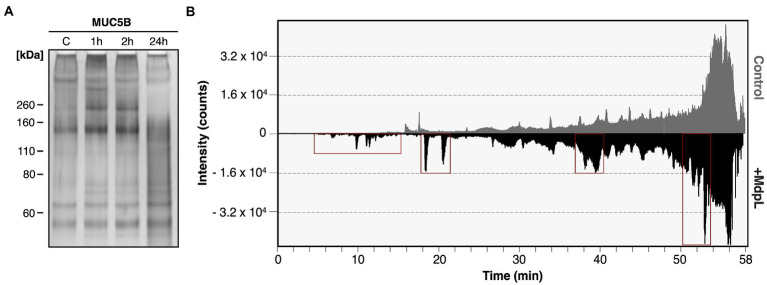
Degradation of MUC5B by MdpL. **(A)** Reduced MUC5B subjected to SDS-PAGE followed by silver staining, displaying low molecular weight fragments after 1, 2, and 24 h incubation with MdpL. **(B)** A mirrored nano-UHPLC chromatogram of digested MUC5B displays the total ion chromatogram (TIC) of Lys-C-digested MUC5B control (upper) and MdpL/LysC-digested MUC5B (lower). Distinct peak increases between the upper and lower panel have been depicted with red boxes.

**Figure 5:**
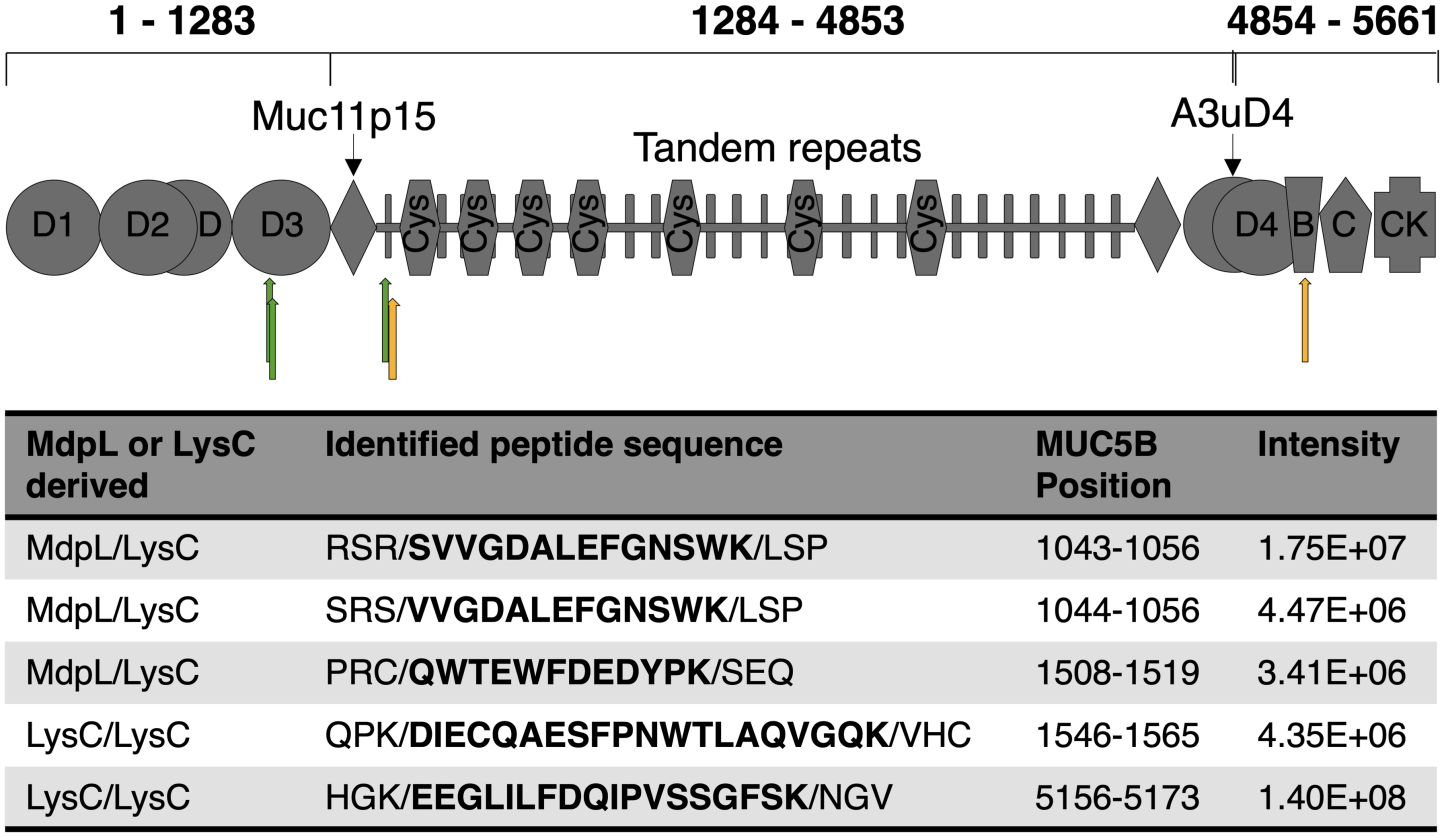
Overview of MdpL hydrolysis on MUC5B. Green and yellow arrows mark the hydrolysis of MdpL/Lys-C, and Lys-C/Lys-C, respectively, subjected to LC–MS/MS, as shown on a schematic figure of main MUC5B domains adapted from Zaretsky and Wreschner (2013a). D1-4: Dimerization domains, von Willebrand factor-like (vWF) containing N-glycosylation sites; Muc11p15: A cysteine and proline-rich peptide conserved in other gel-forming mucins; Tandem repeats: Domain rich in PST-repeat, generally containing a vast amount of O-glycosylation sites; Cys: Cysteine-rich (~10%) subdomain; A3uD4: Domain located between A3 and D4 in vWF; B: vWF type B domain; C: vWF type C domain; CK: Cysteine knot, strictly conserved in all gel-forming mucins. The table depicts the identified peptide sequences resultant to the enzymatic hydrolysis seen after 24 h incubation.

The ability to degrade MUC5B was evaluated by two different methods. The first MUC5B degradation experiment was analyzed with electrophoresis after incubation with MdpL for 1, 2, and 24 h, whereas the MUC5B control was incubated for 24 h. With increased incubation time, low molecular weight fragments appeared in an SDS-PAGE. After 24 h digestion, most bands showed up as a glycoprotein smear with a molecular weight under 200 kDa, and the low band intensity at the top of the well indicated reduced amounts of intact MUC5B as another sign of MUC5B degradation (Figure 4A). In addition, MdpL was shown to degrade MUC5B fragments identified using Western Blotting (Supplementary Figure 9). Reduced MUC5B pre-incubated with MdpL, followed by a sequential hydrolysis by K-specific Lys-C, was subjected to an LC analysis. The displayed number of peaks and the intensity alterations comparing the mirrored chromatograms, indicated MUC5B degradation (Figure 4B). An MS/MS analysis of the same sample subsequent to the LC, led to the identification of unique MUC5B-peptides generated by the enzyme combination. Three MdpL-derived peptides were identified, which suggested no specific amino acid preference (Figure 5). The two identified peptides resulting from hydrolysis by Lys-C solely, were found nearby the MdpL sites in the N-terminal part-, but also in the B domain of MUC5B. Not a single peptide could be found in the control sample.

### MdpL prefers N-terminal digestion of hydrophobic amino acids

**Figure 6:**
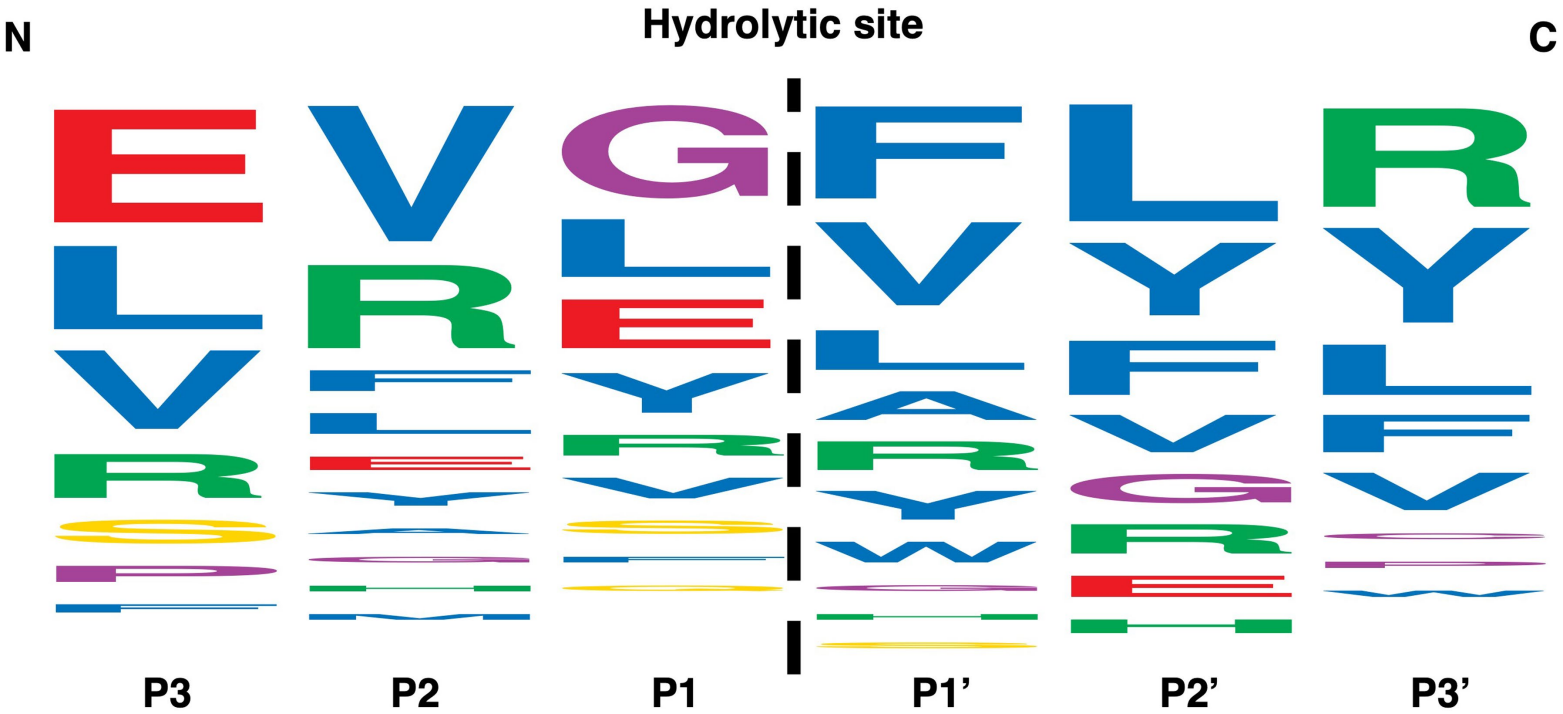
Amino acid specificity analyzed by LC-MS/MS. The position and size of each letter is based on the frequency of a particular amino acid +/− three positions from the MdpL hydrolytic site (dashed line). The larger the size in a particular position, the more frequent the amino acid appears after digestion of Neuromedin-U25, MOG, and Insulin Chain B Oxidized, which was based on the 89 identified peptides from the hydrolyses. Amino acids are given a color based on the side chain; Red – negative charge, Green – positive charge, Yellow – uncharged, Purple – special, Blue – hydrophobic.

Seven peptides with a difference in amino acid composition and size were selected to investigate the specificity of MdpL (Figure 6). Four of the peptides, Drosocin, H2686, H8390, and Amyloid β-Protein, all characterized by an amino acid chain length of less than 20, were not hydrolyzed by MdpL. Neuromedin-U25, MOG, and Insulin Chain B Oxidized were hydrolyzed at multiple sites and the generated high score peptides in LC–MS/MS analyzes were entered into WebLogo, which ordered the amino acids from top to bottom by their prevalence in a specific position from the digestion site between P1 and P1’ (Figure 6). MdpL seemed to be promiscuous in the sense that it has no clear amino acid preference, although a high number of amino acids with hydrophobic side chains were detected proximally to the C-terminal at the hydrolytic site. Other types of amino acids were still prevalent in the same position, but the data were suggestive of MdpL having a hydrophobic trait preference. The base peak chromatogram, all identified peptides, and a digestion overview from the Neuromedin-U25, MOG, and IOB reactions were assembled and interpreted to obtain a detailed picture of the MdpL preference (Supplementary Figure 10). These data served as the basis for this specific WebLogo construction.

### MdpL is identified in cell-associated samples of *Limosilactobacillus fermentum* culture

**Figure 7:**
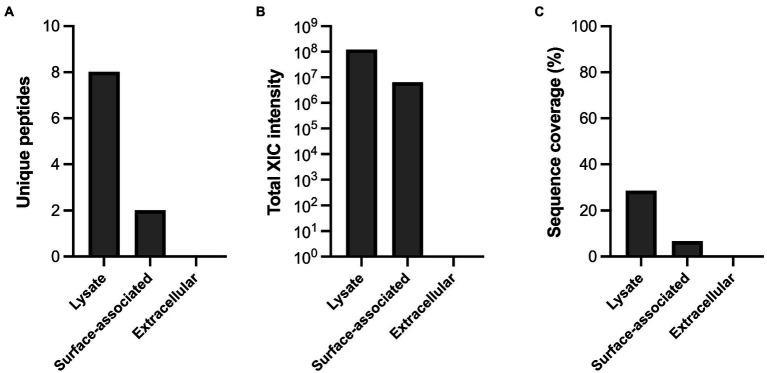
Cellular localization of MdpL in *Limosilactobacillus fermentum*. **(A)** The quantity found in either lysate, surface-associated or extracellular samples of *L. fermentum* is calculated and based on the identified unique MdpL-derived peptides, **(B)** the total extracted ion current (XIC) intensity from MdpL peptides **(C)** and the total sequence coverage of all MdpL peptides.

The cellular location of MdpL was investigated by separating a *L. fermentum* culture into cellular and secreted fractions, hydrolyzing the proteins with trypsin, followed by LC–MS/MS analysis (Figure 7). The cellular fraction was treated by sonication or papain and was thereafter annotated as lysate and surface-associated fractions, respectively, and these were the only fractions containing unique MdpL peptides (Figure 7A). The total extracted ion currents were measured, where the lysate had an intensity of 10^8^, the surface-associated fraction about 10^7^, and the extracellular gave a null result (Figure 7B). The differences in sequence coverage resembled the stack pattern seen with the unique peptides, suggesting that MdpL is localized in close association to the cell, either intracellularly or in close proximity to the cell surface (Figure 7C).

## Discussion

The role of the protease producing bacterium *L. fermentum* in multispecies dental biofilms is not well known. When a clinical isolate, *L. fermentum* MAL1, was grown in a MUC5B biofilm environment an apparent protease presence was observed, and compared to a nutrient broth environment a 2.7-fold increase was seen for a putative O-sialoglycoprotein endopeptidase (Wickström et al., 2013). In this study, we have identified and characterized a protease, MdpL, which hydrolyses substrates with mucin-like features independently of O-glycans and is highly conserved in commensal oral bacteria.

The homology study confirmed MdpL as an important enzyme for *L. fermentum* on a species level, with a high primary structure conservation, except for the N-terminal part which was not present in all strains (Figure 1). The low number of amino acid mutations indicates that the hydrolytic function is not significantly affected and suggests that the same enzymatic features are kept across the species. Furthermore, the high conservation of the MdpL within *L.* fermentum indicate a key enzyme for survival in natural habitats. Given the specificity of the enzyme favoring MUC5B compared to other substrates, we foresee that this is the main substrate for MdpL. The effect of such hydrolysis could certainly impact both nutrition, adherence and general fitness in its habitat. High similarity to the disease-associated species *S. mutans* (Forssten et al., 2010), *S. pneumoniae* (van der Poll and Opal, 2009), and *G. vaginalis* (Morrill et al., 2020) does not necessarily imply that the enzyme is a virulence factor. These bacteria share a key feature for attachment and nutrition: They all live in a mucosal environment with MUC5B as the predominant glycoprotein. Complementing this with the fact that MdpL appears to be a shared feature among commensals of the oral cavity which typically are associated with bacterial eubiosis, points at a common enzymatic mucin degradation function, i.e., sourcing carbon and nitrogen for nutritional purposes (Derrien et al., 2004). This suggests that MdpL and MdpL-like enzymes are important to sustain an eubiotic oral environment.

The characterization confirms that MdpL depends on a reducing environment, similarly to other proteases expressed in the dental plaque, i.e., RgpB from *P. gingivalis*. This suggests that *L. fermentum* has a system that supports the bacterium in maintaining an appropriate reduction level, which will ensure proper protein folding but should also increase the enzymatic activity. L-cystine, the disulfide dimer of L-cysteine, of huge importance for MdpL’s activity, is available to dental plaque bacteria from the protein subfraction of saliva (Cowman et al., 1977). The surface-exposed cystine-binding-protein A (BspA) from *L. fermentum* BR11, as part of the ATP-binding cassette (ABC)-type cystine transportation system, is indispensable for the establishment of a reduced environment (Turner et al., 1999; Hung et al., 2005). BspA is pivotal to sequester L-cystine, which is intracellularly reduced to L-cysteine. Moreover, L-cysteine is important for the accurate operation of the glutathione redox system (GST), which is another key component in the regulation of a reduced cellular environment (Zhang and Forman, 2012). A study involving the CECT 5716 strain of *L. fermentum*, has demonstrated how the bacterium can utilize GST (Surya et al., 2018). Additionally, the *L. fermentum* ME-3 strain has been found to have a complete GST system, including uptake, synthesis, and redox turnover, and hence it can serve as an antioxidative apparatus retaining a reduced environment (Kullisaar et al., 2010), implying that *L. fermentum* strains have a complex strategy for the proper expression of proteases such as MdpL.

Our analysis revealed that the *L. fermentum* genome lacks the genes for oligopeptide transport system (Opp) homologs. The Opp system manages internalization of oligopeptides in the 4–8 amino acid range (Kunji et al., 1996), which are then further degraded by intracellular peptidases. Typically, the oligopeptides are released by the caseinolytic enzymes PrtP, PrtB, and PrtH, which are commonly expressed by *Lactobacillaceae* (Kunji et al., 1996), but Prt-like enzymes have not been identified and described in *L. fermentum* (Wickström et al., 2013). This indicates that *L. fermentum* could have a significantly different transport system, which could allow larger peptides to enter the bacterial cell, as described for *Streptococcus thermophilus* (Rodríguez-Serrano et al., 2018). Considering the peptide length and high glycan content seen after MUC5B hydrolysis by MdpL, further hydrolysis of mucin by other yet unidentified enzymes before the peptides are entering the cell could be an alternative for nutrient uptake. This hypothesis is supported by the MdpL peptide preference, where only three out of the seven peptides incubated with MdpL, were hydrolyzed (Figure 6; Supplementary Figure 10). The four undigested peptides all included hydrophobic amino acids, suggesting that MdpL should be able to hydrolyze them. However, the predominant feature of the non-hydrolyzed substrates is the peptide length, being shorter than 20 amino acids. Further, the results suggest a strong preference for peptides longer than 20 amino acids, but if hydrophobic amino acids dominate smaller peptides (10–20 aa) such as the partly degraded MOG peptide, MdpL will also hydrolyze those to some extent. This size preference of MdpL hints at a hitherto unrecognized proteolytic system of *L. fermentum*.

Investigations of the cellular location of MdpL showed that the enzyme is located in both lysate and surface-associated fractions of a *L. fermentum* culture (Figure 7). MdpL has been suggested to be located on the outside of the biofilm resident *L. fermentum* cell membrane (Wickström et al., 2013), emphasizing that the enzyme is surface-associated. It is however unclear how the enzyme is attached to the surface due to the lack of an annotated signal peptide in the protein sequence. Other proteins annotated as cytoplasmic have through proteomics studies been identified in the secretome and a non-canonical secretion pathway have been hypothesized for these (Siciliano et al., 2019). The extracellular location could be of hydrolytic benefit, given the close proximity to mucin. It should be noted however that attempts to measure activity of native enzyme has not been successful. *Limosilactobacillus fermentum* BCS87 has been shown to express a mucin-binding protein, 32-Mmubp on the cell surface, facilitating adherence to piglet mucus and porcine mucin *in vitro* (Macías-Rodríguez et al., 2009). The MBD_93_ domain of MucBP, another mucin-binding protein from *L. fermentum*, was suggested to have high affinity for the typical mucin glycans N-acetylgalactosamine, N-acetylglucosamine, galactose, and sialic acid (Chatterjee et al., 2018). If we assume that these proteins are conserved within the species, they could be beneficial to MdpL by providing it with direct access to its natural substrate.

The proposition that MUC5B probably is the natural substrate of MdpL is supported by the results from the substrate specificity tests (Figure 3). The activity toward the tested O-glycoprotein substrates, suggests that the higher the similarity to MUC5B in terms of glycosylation, the higher is the activity. Unexpectedly, in regard to the putative *in silico* O-sialo specificity, MdpL does not require adjacent O-glycans to hydrolyze the protein backbone. When all O-glycan moieties were removed from Abatacept, Etanercept, and bovine fetuin, MdpL was still able to hydrolyze the substrates. The two former substrates are similar in terms of structure and hydrolytic access but differs in terms of O-glycan occurrence (Etanercept has more O-glycans), suggesting that they should be hydrolyzed to a similar extent. However, Abatacept is only partly hydrolyzed, whereas Etanercept is almost completely degraded. Complementing these results with the comprehensive hydrolysis of MUC5B (Figures 4, 5), accentuates the hypothesis that MUC5B is MdpL’s natural substrate. Lys-C cannot hydrolyze MUC5B without MdpL pre-incubation, indicating that MdpL has opened up the previously inaccessible structure, thus enabling Lys-C hydrolysis. The sequential degradation of MUC5B further confirms the pronounced activity toward the large glycoprotein. The migration pattern seen for MUC5B (Figure 4A) is due to fragmentation of MUC5B during sample preparation. Hence, we cannot be certain if MdpL is initiating the MUC5B hydrolysis or if the enzyme only hydrolyzes fragmented MUC5B. However, hydrolysis activity at the N-terminus of the mature MUC5B could potentially disrupt the gel-forming properties of the mucin with implications for the surrounding environment. The structural complexity of mucin-derived O-glycopeptides, which may carry hundreds of monosaccharides, could exclude interesting peptides from the analysis. The instrumentation used, and the parameters applied did not allow detection of such complex O-glycopeptides; hence it cannot be ruled out that MdpL also hydrolyses in the region of O-glycosylated tandem repeats.

Even though the observed amino acid specificity of MdpL may be biased by the use of peptides with a high prevalence of certain amino acids, it is reasonable to propose that MdpL prefers hydrophobic amino acids (Figure 6). Complementing these results with the hydrolytic sites detected on MUC5B, suggests that MdpL has similarities to the calpain family of proteases. Those enzymes have been found to cleave the von Willebrand factor (vWF) in circulating blood (Furlan et al., 1996), which is interesting in a mucin degrading perspective, since MUC5B consist of four vWF-like domains in its N-terminal part, as depicted in the schematic figure of MUC5B (Figure 5). Calpains typically hydrolyze proteins if Leu, Phe, Tyr, or Val is in the P1 position (Cuerrier et al., 2005), which closely resembles the MdpL preferences, except that MdpL have these amino acids on the C-terminal side (P1’) of its preferred hydrolytic site. Another MdpL-resembling feature of calpains is their association with the cell membrane, occurring after Ca^2+^ activated translocation from the cytoplasm (Sakai et al., 1989; Gil-Parrado et al., 2003).

In future studies, the highly conserved nature of MdpL should be considered. The homology seen across bacterial species does not necessarily indicate a high sequence similarity, and amino acids located in the enzyme’s active site could be replaced in the other species, which could influence the function. Furthermore, this conserved enzyme group has increased the knowledge on mucin degradation and complex biofilm dynamics, but their involvement in nutrient acquisition within the oral biofilm needs further consideration.

In conclusion, the role of *L. fermentum* in establishing an eubiotic oral environment is accentuated in this study of a new protease, MdpL from *L. fermentum*, with a focus on the activity on human salivary MUC5B.

## Data availability statement

The mass spectrometry proteomics data have been deposited to the ProteomeXchange Consortium *via* the PRIDE (Perez-Roverol et al., 2021) partner repository with the dataset identifier PXD039681.

## Author contributions

FL, GS, RL, and CW conceived and designed the study, analyzed the data, and prepared the manuscript. FL carried out the experiments. All authors contributed to the article and approved the submitted version.

## Funding

Open access funding provided by Malmö University. The authors acknowledge financial support from Genovis AB, Lund, Sweden. FL is part of the Combine Graduate Research School at Malmö University funded by the KK Foundation, Grant No. 202100-4920.

## Conflict of interest

FL was employed by Genovis AB.

The remaining authors declare that the research was conducted in the absence of any commercial or financial relationships that could be construed as a potential conflict of interest.

## Publisher’s note

All claims expressed in this article are solely those of the authors and do not necessarily represent those of their affiliated organizations, or those of the publisher, the editors and the reviewers. Any product that may be evaluated in this article, or claim that may be made by its manufacturer, is not guaranteed or endorsed by the publisher.
