# Supplementary material for: Characterization of a highly conserved MUC5B-degrading protease, MdpL, from Limosilactobacillus fermentum
Source: Front Microbiol. 2023 Feb 28;14:1127466. doi: 10.3389/fmicb.2023.1127466 (PMC10011156; doi:10.3389/fmicb.2023.1127466)
Supplement: Supplementary file 1 [file Data_Sheet_1.PDF]

Supplementary Material

Characterization of a highly conserved MUC5B-degrading protease, MdpL, from *Limosilactobacillus fermentum*

Fredrik Leo[*]**, Gunnel Svensäter, Rolf Lood, Claes Wickström**

*** Correspondence:** Fredrik Leo: fredrik.leo@mau.se

Supplementary table 1: List of bacterial species included in the sequence homology study.

**Bacterial species**	**GenBank accession number**
Limosilactobacillus fermentum	WP_057727250.1
Actinomyces naeslundii	WP_076255305.1
Actinomyces sp.	EEZ78368.1
Actinomyces urinae	WP_072344819.1
Aggregatibacter actinomycetemcomitans	WP_179218506.1
Akkermansia muciniphila	WP_179218506.1
Bifidobacterium callitrichios	WP_107043203.1
Campylobacter jejuni	EDB0641308.1
Candida albicans	KHC51017.1
Corynebacterium matruchotii	WP_005519844.1
Escherichia coli	MBO9150451.1
Fusobacterium nucleatum	WP_098997538.1
Gardnerella vaginalis	PMC27815.1
Helicobacter pylori	WP_128030481.1
Limosilactobacillus mucosae	WP_074504505.1
Limosilactobacillus oris	AMS08677.1
Limosilactobacillus reuteri	WP_086120334.1
Parvimonas micra	WP_029950383.1
Porphyromonas gingivalis	WP_058018687.1
Rothia dentocariosa	MBF1647186.1
Streptococcus anginosus	WP_195323402.1
Streptococcus constellatus	WP_119055688.1
Streptococcus gordonii	WP_046164911.1
Streptococcus mutans	WP_019318710.1
Streptococcus oralis	WP_084852800.1
Streptococcus pneumoniae	CWH93831.1
Streptococcus sanguinis	WP_072073344.1
Streptococcus sobrinus	WP_019785239.1
Treponema denticola	WP_002688239.1
Veillonella parvula	MBS4892419.1

>MdpL
MAFESSCDETSVAVIEDGHRVLSNIVATQIASHQRFGGVVPEVASRHHIEQITKCTKEALEQA
GVSYQDLTAVAVTYGPGLVGSLLIGVTAAKTIAWAHQLPLVPVNHMAGHLYAARFVSDFTY
PMLGLLVSGGHTELVYMKEEHDYQIIGETRDDAAGEAYDKVGRVMGINYPAGKTVDQWAA
KGHDTFHFPRAMEKEDNFDFSFSGLKSAFINTVHNADQRGEVLDKYDLAASFQQSVVDVLV
AKTIRALDEYPVKQLILAGGVAANQGLRKQLSASLQAKHPEVQLLQAPLKYCGDNAAMIGA
AGYVNYLHGDRADGSLNAVPGLSFAHLKEENGSGHHHHHH

Supplementary figure 1: FASTA sequence of MdpL (GenBank accession number KRN17993.1) with C-terminal GSG and 6xHis-tag.

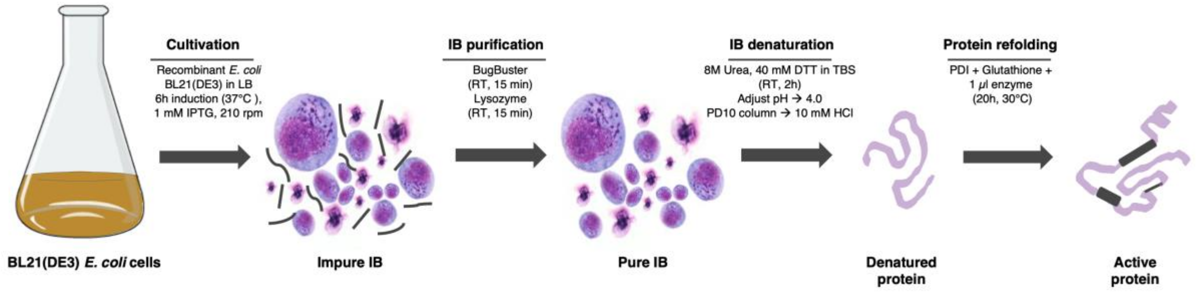

Supplementary figure 2: Flowchart of transformed *E. coli* cultivation, inclusion body (IB) formation, purification, and denaturation, and finally MdpL refolding by Protein Disulfide Isomerase, PDI, and reduced/oxidized glutathione.

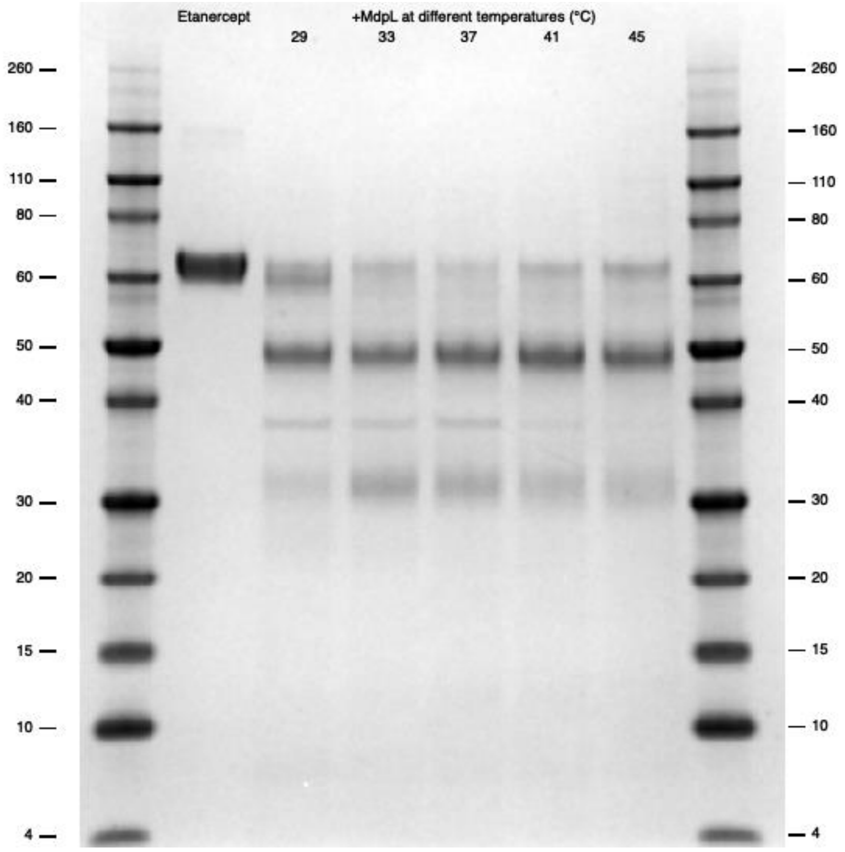

Supplementary figure 3: A representative gel assessing the effect of temperature on MdpL activity.

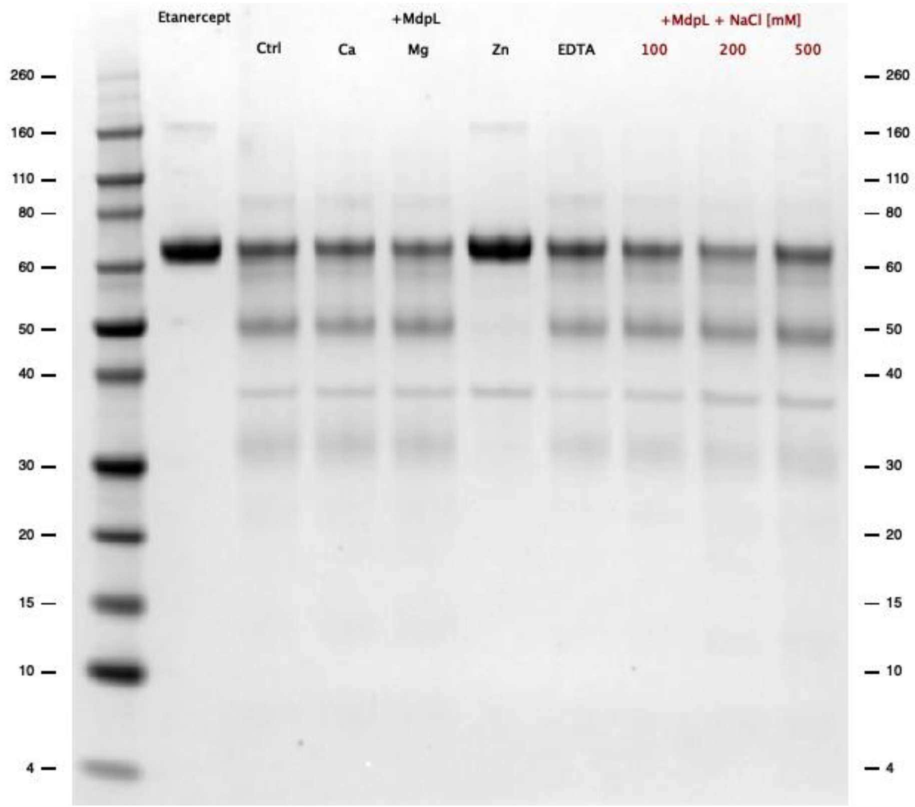

Supplementary figure 4: A representative gel assessing the effect of ions, EDTA and NaCl on MdpL activity.

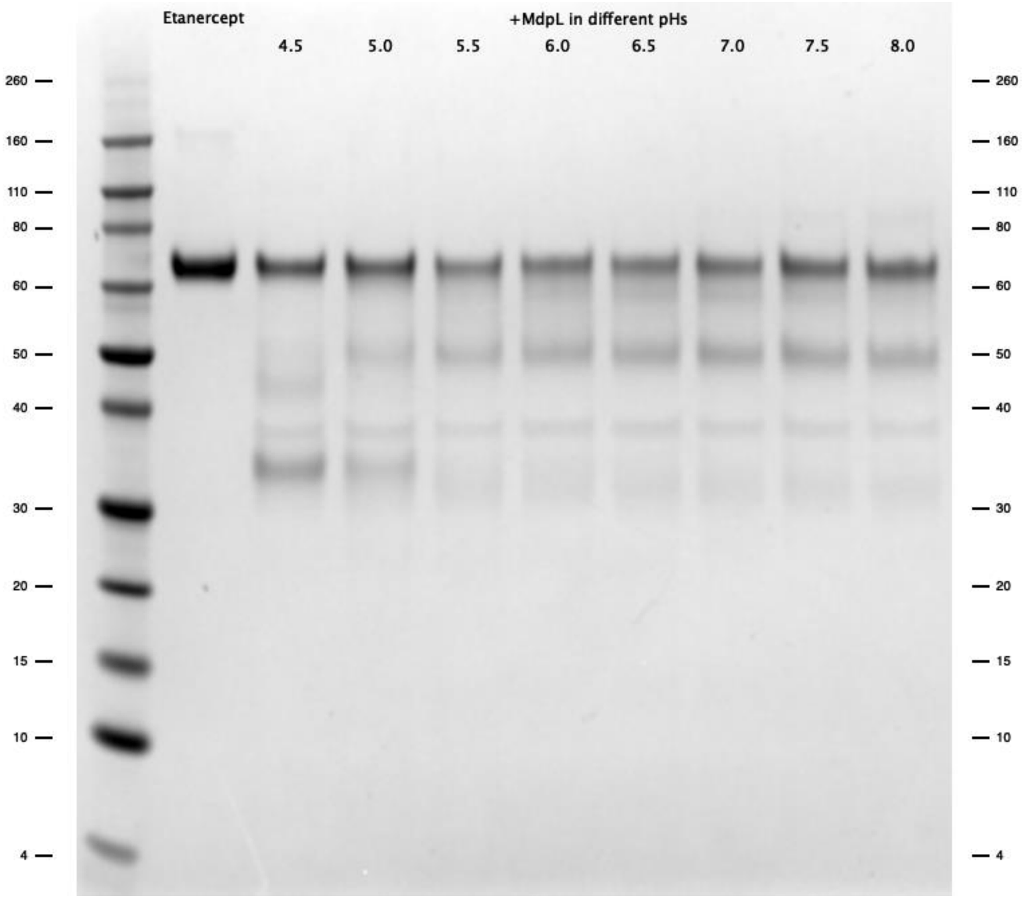

Supplementary figure 5: A representative gel assessing the effect of pH on MdpL activity.

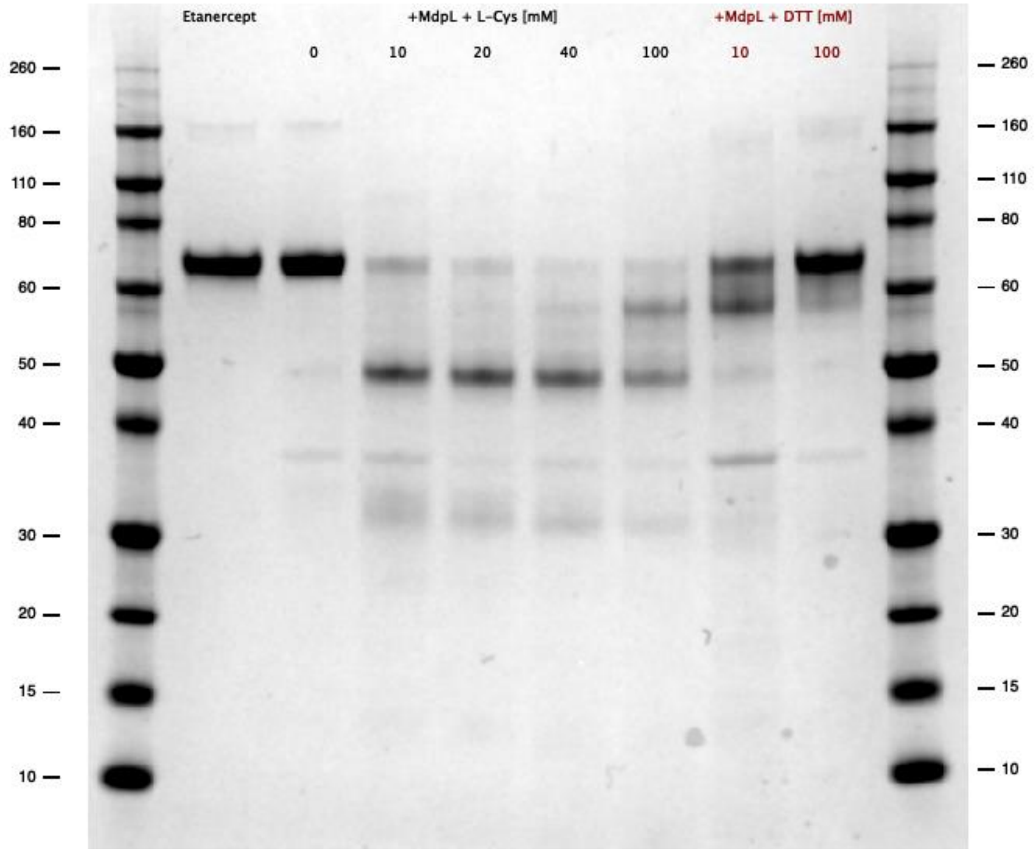

Supplementary figure 6: A representative gel assessing the effect of reducing agents L-cys and DTT on MdpL activity.

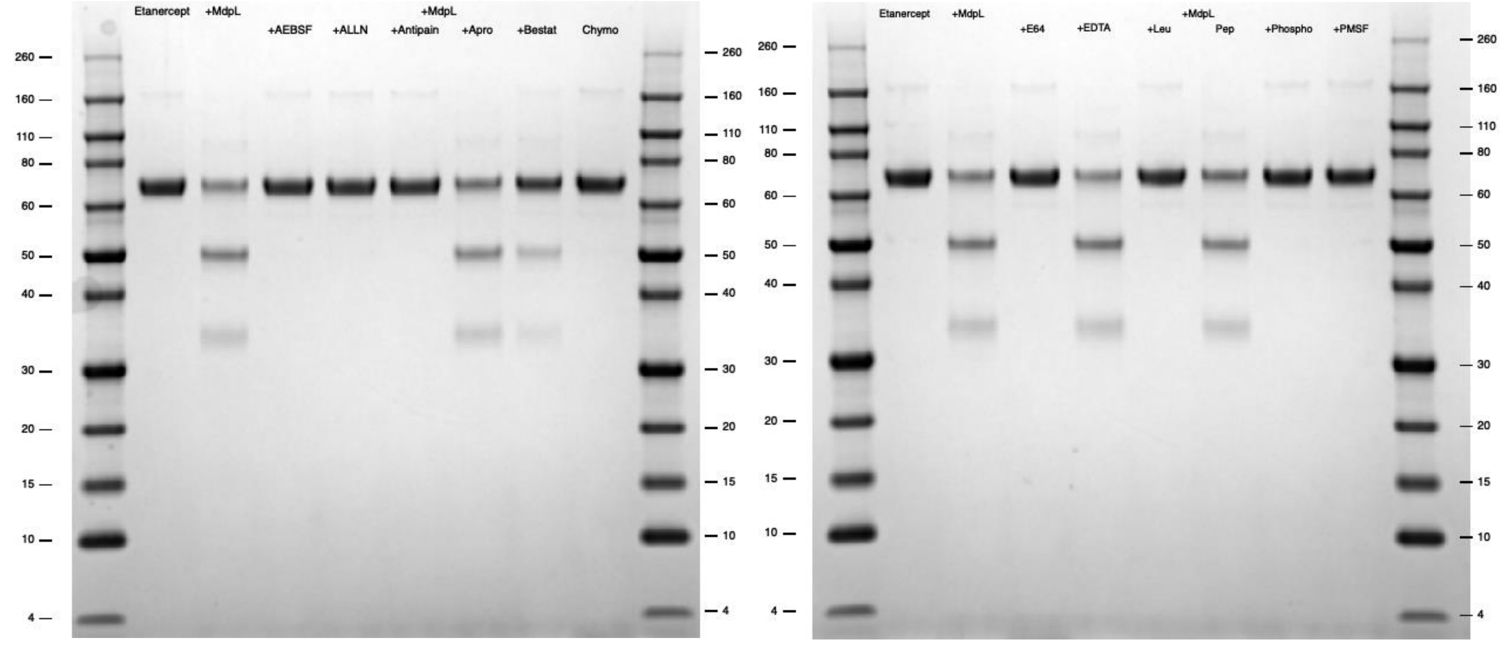

Supplementary figure 7: A representative gel assessing the effect of protease inhibitors on MdpL activity.

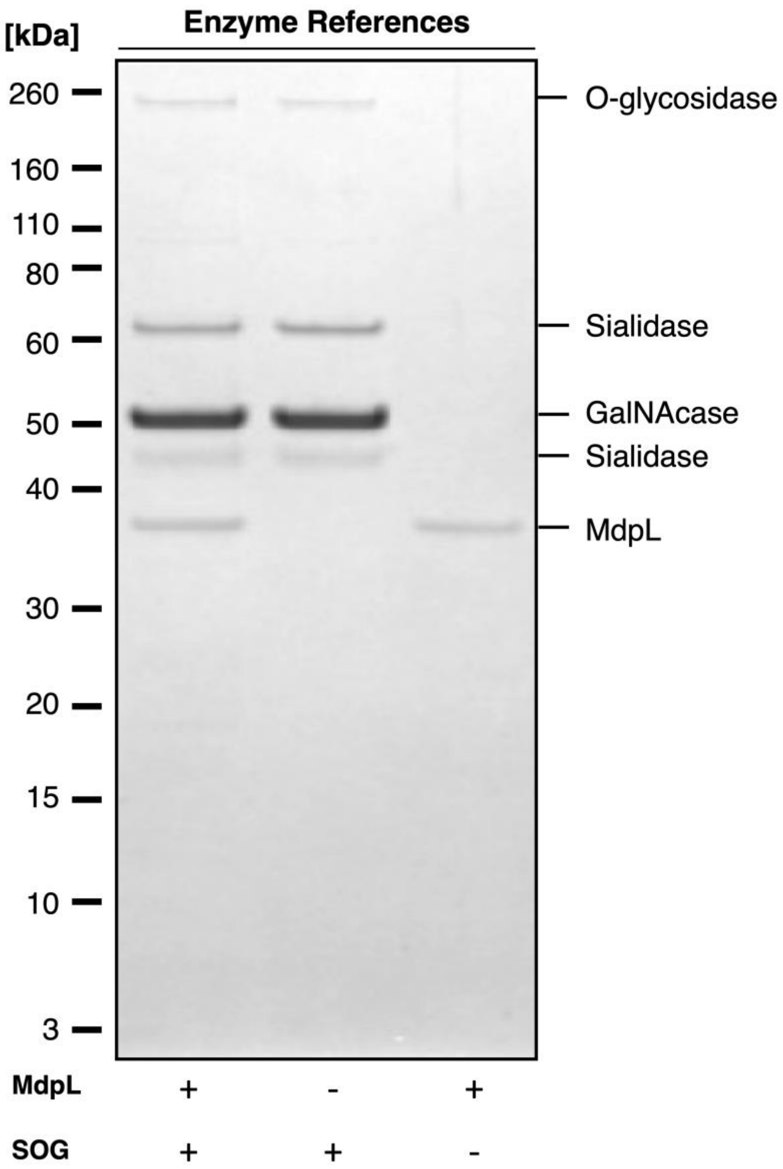

Supplementary figure 8: MdpL and SOG reference bands as used for the proteolytic activity tests.

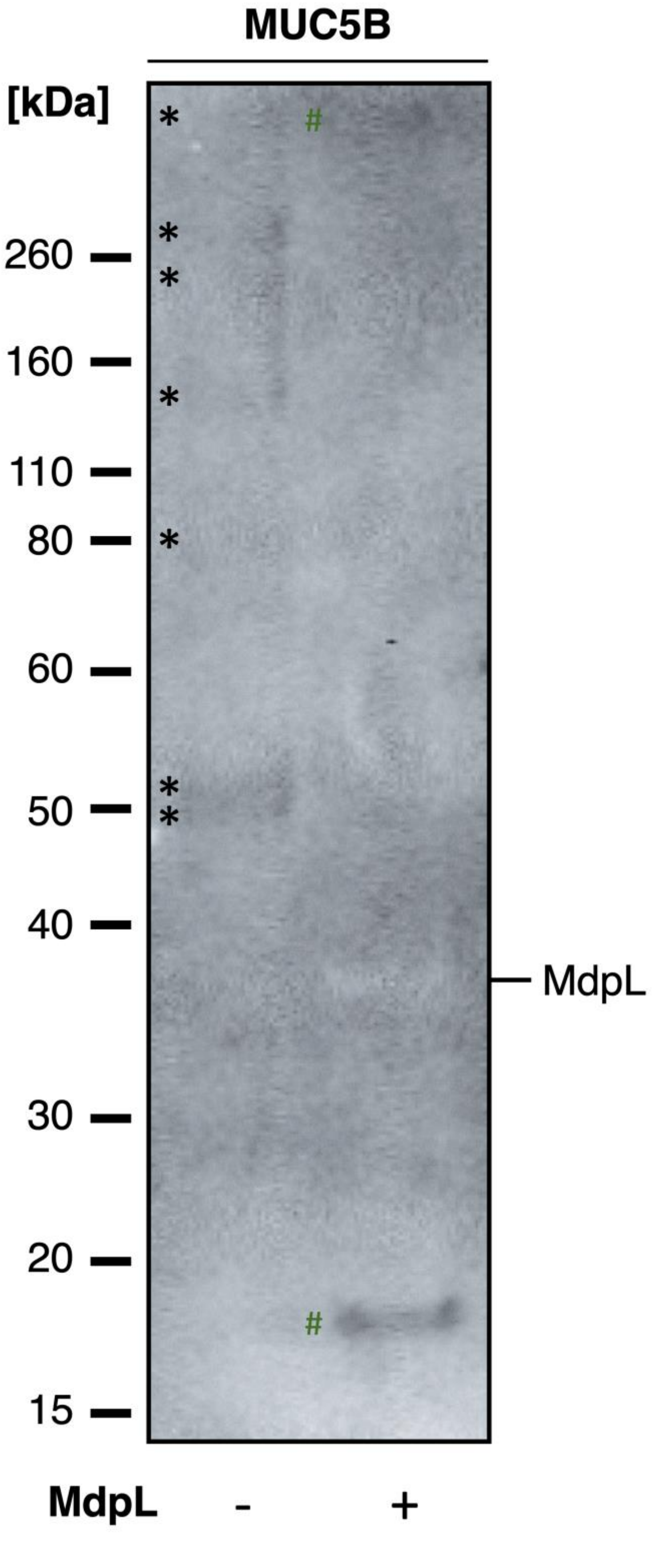

Supplementary figure 9: Degradation of MUC5B fragments by MdpL. Reduced MUC5B subjected to Western blot analysis, displaying hydrolysis of MUC5B fragments after 24 h incubation with MdpL. Intact (*), and MdpL-generated MUC5B fragments (#).

A

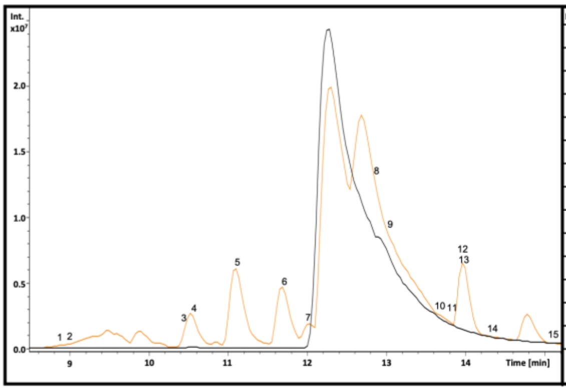

Peptide #	Range (aa)	Mr calc.	Mr meas.	Δ Mr [ppm]	TIC Intensity	Retention time
1	14-25	1665.8964	1665.8913	-2.35	8.179E+04	8.82
2	19-25	948.5294	948.5253	-4.28	1.341E+04	8.96
3	3-17	1772.8806	1772.8720	-4.80	8.432E+04	10.43
4	18-25	1111.5927	1111.5876	-4.60	4.630E+05	10.53
5	1-17	2048.0439	2048.0358	-3.03	2.214E+06	11.12
6	1-18	2211.1073	2211.1038	-3.03	6.005E+06	11.70
7	5-25	2652.3673	2652.3772	3.74	1.160E+05	11.97
8	1-10	1194.5557	1194.5529	-2.36	8.420E+05	12.83
9	1-19	2358.1757	2358.1678	-3.34	1.729E+06	13.00
10	1-20	2471.2597	2471.2544	-3.91	3.137E+06	13.62
11	1-15	1735.8417	1735.8372	-2.61	5.131E+05	13.83
12	1-13	1493.7402	1493.7334	-3.27	6.615E+06	13.96
13	1-14	1621.7988	1621.7907	-4.99	2.722E+05	14.00
14	1-12	1406.7082	1406.7039	-3.03	1.805E+05	14.34
15	3-21	2343.1648	2343.1565	-3.52	3.877E+04	15.09

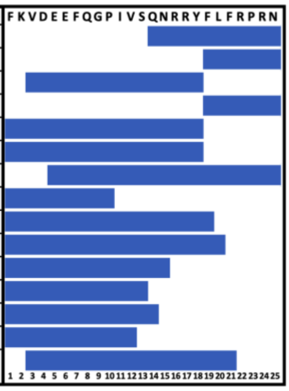

B

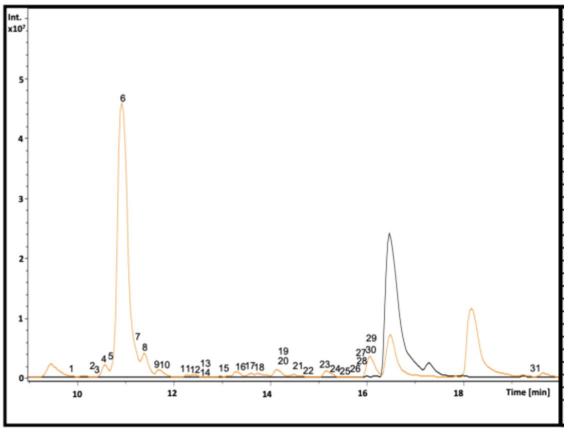

Peptide #	Range (aa)	Mr calc.	Mr meas.	Δ Mr [ppm]	TIC Intensity	Retention time
1	5-14	1112.4921	1112.4966	4.12	1.770E+04	9.89
2	1-8	964.4073	964.4094	2.17	9.679E+04	10.27
3	20-30	1271.6299	1271.6316	1.37	3.522E+04	10.37
4	1-11	1301.5823	1301.5881	4.47	4.219E+05	10.54
5	24-29	801.4061	801.4072	1.37	2.140E+06	10.61
6	24-30	872.4432	872.4461	3.24	4.586E+07	10.95
7	16-23	943.4069	943.4097	2.92	3.041E+05	11.12
8	1-13	1529.6933	1529.7016	5.47	5.202E+06	11.39
9	1-14	1600.7304	1600.7410	5.85	3.155E+05	11.60
10	23-30	929.4647	929.4688	4.41	2.549E+05	11.70
11	19-30	1422.6238	1422.6342	7.34	3.249E+04	12.15
12	18-30	1521.6922	1521.7030	7.11	4.395E+05	12.29
13	1-13*	1530.6773	1530.6896	8.07	1.506E+05	12.56
14	5-15	1225.5761	1225.5841	6.51	3.970E+04	12.63
15	17-30	1634.7763	1634.7837	6.25	2.773E+05	13.00
16	14-23	1127.5281	1127.5335	4.79	1.035E+06	13.31
17	14-22	1070.5066	1070.5117	4.71	2.067E+04	13.48
18	1-15	1713.8145	1713.8267	7.17	1.011E+06	13.58
19	16-30	1797.8396	1797.8519	7.90	1.808E+06	14.17
20	9-23	1692.8141	1692.8277	7.99	9.193E+04	14.17
21	12-23	1355.6391	1355.6505	8.42	2.145E+05	14.54
22	1-16	1876.8778	1876.8938	9.58	4.312E+05	14.68
23	15-30	1910.9237	1910.9381	8.16	2.729E+05	15.09
24	14-30	1981.9608	1981.9763	8.77	1.899E+06	15.19
25	13-30	2111.0034	2111.0174	7.52	1.161E+05	15.50
26	4-23	2279.0311	2279.0503	7.80	1.145E+05	15.60
27	5-23	2150.9725	2150.9908	8.88	1.081E+05	15.71
28	12-30	2210.0718	2210.0901	9.00	3.360E+05	15.77
29	14-24	1274.5965	1274.6063	7.64	3.292E+04	15.88
30	1-17	1989.9618	1989.9788	8.52	2.649E+05	16.01
31	1-19	2240.0242	2240.0381	6.67	3.591E+04	19.40

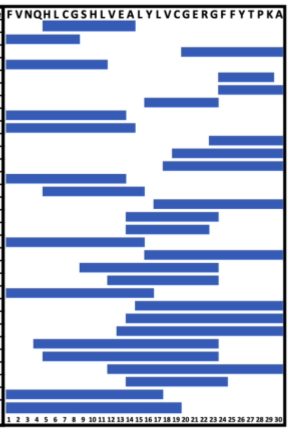

C

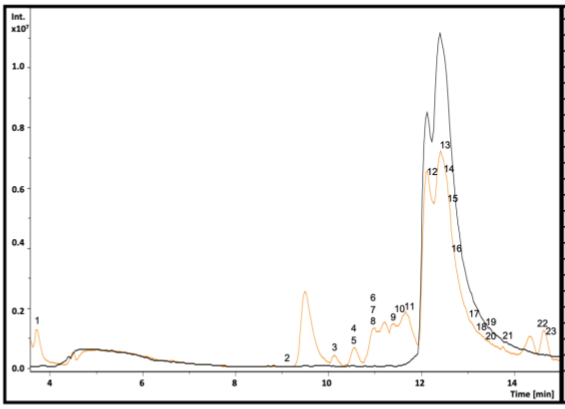

Peptide #	Range (aa)	Mr calc.	Mr meas.	Δ Mr [ppm]	TIC Intensity	Retention time
1	1-4	434.1835	434.1832	-0.70	2.381E+04	3.84
2	7-21	1825.0224	1825.0253	1.62	3.300E+03	9.07
3	6-21	1989.0697	1989.0887	8.19	4.311E+04	10.20
4	5-12	1107.5613	1107.5636	-7.13	3.124E+05	10.57
5	5-15	1442.7571	1442.7612	1.91	1.048E+06	10.57
6	5-21	2174.1650	2174.1719	1.53	2.139E+06	10.98
7	3-15	1598.8470	1598.8522	1.61	1.97E+06	11.12
8	5-13	1206.6298	1206.6314	1.36	1.221E+05	11.19
9	3-21	2330.2549	2330.2602	1.03	2.160E+06	11.40
10	5-18	1875.0056	1874.9995	-3.25	3.306E+05	11.60
11	3-21	2331.2389	2331.2577	5.51	2.886E+06	11.63
12	1-15	1858.9301	1858.9363	1.54	4.630E+06	12.22
13	5-11	951.4603	951.4627	2.50	1.362E+05	12.49
14	1-9	1133.5328	1133.5369	3.61	1.184E+05	12.66
15	1-12	1523.7344	1523.7392	-0.92	3.576E+05	12.76
16	1-18	2291.1786	2291.1864	3.42	7.483E+05	12.90
17	1-13	1622.8028	1622.8078	3.08	1.027E+06	13.07
18	3-11	1107.5502	1107.5535	3.01	2.406E+05	13.35
19	1-14	1721.8712	1721.8754	2.48	1.915E+05	13.55
20	1-16	1972.0142	1972.0205	1.70	5.269E+05	13.55
21	1-17	2135.0775	2135.0828	1.08	5.541E+05	13.93
22	1-11	1367.6332	1367.6373	2.94	1.291E+06	14.65
23	1-10	1280.6012	1280.6046	2.63	3.095E+04	14.78

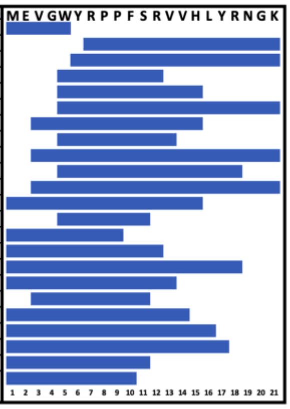

Supplementary figure 10: LC-MS^2 data from Neuromedin (A), IOB (B), and MOG (C) peptide digestion by MdpL. The left section in A, B, and C represents the base peak chromatogram with (orange) or without MdpL (black). The numbers above the lines of the chromatogram corresponds to a MdpL-generated peptide (score >30) found in the middle section. The right section illustrates the digestion pattern of MdpL and is a summary of all the peptides found. The peptide amino acid sequence and -number, are found above and below the blue boxes.
